# Rescue of Transgenic Alzheimer’s Pathophysiology by Polymeric Cellular Prion Protein Antagonists

**DOI:** 10.1016/j.celrep.2019.01.064

**Published:** 2019-01-29

**Authors:** Erik C. Gunther, Levi M. Smith, Mikhail A. Kostylev, Timothy O. Cox, Adam C. Kaufman, Suho Lee, Ewa Folta-Stogniew, George D. Maynard, Ji Won Um, Massimiliano Stagi, Jacqueline K. Heiss, Austin Stoner, Geoff P. Noble, Hideyuki Takahashi, Laura T. Haas, John S. Schneekloth, Janie Merkel, Christopher Teran, Zahra K. Naderi, Surachai Supattapone, Stephen M. Strittmatter

(Cell Reports 26, 145–158.e1–e8; January 2, 2019)

In the originally published version of this article, author name Zahra K. Naderi was misspelled in the author list. This has now been corrected.

The authors regret this error.

